# Process optimization of microwave drying for rice based on response surface methodology

**DOI:** 10.1371/journal.pone.0340356

**Published:** 2026-01-13

**Authors:** Chunshan Liu, Kezhen Chang, Jie Li, Jinquan Li, Siyu Chen, Yi Jin, Zhongjie Zhang, Zhigao Tang

**Affiliations:** 1 College of Mechanical Engineering, Jiamusi University, Jiamusi, China; 2 Academy of National Food and Strategic Reserves Administration, Institute of Grain Storage and Logistics, Beijing, China; 3 National Engineering Research Center of Grain Storage and Logistics, Beijing, China; 4 Jiangsu Macrowave Microwave Technology Co., Ltd, Suzhou, China; Shahrekord University, IRAN, ISLAMIC REPUBLIC OF

## Abstract

To optimize the microwave drying process of paddy rice and improve its quality, the effects of hot air temperature, microwave power, and grain layer thickness on the post-drying potassium content, calcium content, vitamin B₁ content, and free fatty acid content of rice were investigated. Using response surface methodology, an experimental scheme based on the Box-Behnken design was constructed to analyze the influence of these three factors on the four nutritional and biochemical indicators. A multi-index optimization model was established and validated. The results showed that all response indicator models were statistically significant. The optimal process parameters were determined as follows: hot air temperature of 52.47°C, microwave power of 20 kW, and grain layer thickness of 2.78 cm. The corresponding predicted values were potassium content of 3724 mg/kg, calcium content of 113.7 mg/kg, vitamin B₁ content of 0.290 mg/100g, and free fatty acid content of 21.5 mg/100g. Validation experiments demonstrated that the dried rice under these conditions exhibited excellent quality, with relative errors between predicted and measured values below 3%, indicating the reliability of the model and the significant improvement in rice drying quality achieved by the optimized process. Furthermore, visual process reference charts were developed to provide a theoretical basis for adjusting process parameters in practical production.

## Introduction

As a staple food crop worldwide, the yield and quality of paddy rice profoundly impact human health [1–3]. In China, due to the relatively lagging development of agricultural modernization, most farmers still rely on traditional natural sun-drying methods post-harvest. These methods suffer from prolonged drying cycles, inefficiency, and susceptibility to weather conditions, thereby challenging the consistency of rice quality [4–5]. Failure to promptly reduce moisture content to safe content can sustain enzymatic activity in rice grains, leading to mold growth and spoilage, underscoring drying as a critical step in post-harvest processing [6–8].

Grain drying is a complex process characterized by its nonlinear, time-delayed, and multivariable-coupled nature. The demand for large-scale and timely drying further imposes stringent requirements on the industrial performance of drying equipment [9]. This process is influenced not only by the physical structure and chemical properties of the material but also by drying conditions and techniques [10]. Microwave drying, widely adopted in industrial applications, leverages microwave radiation to achieve simultaneous internal and external heating of materials [11–13]. Unlike conventional hot air drying, which relies on unidirectional heat transfer from the surface inward, microwave penetration enables balanced moisture gradient mitigation within materials, avoiding the “case-hardening effect” caused by surface hardening [14–15]. Nevertheless, industrial-scale microwave drying faces challenges.The thermosensitive nature of grains under high-temperature, high-humidity conditions necessitates dynamic equilibrium models that balance moisture migration rates with quality preservation [16]. In recent years, researchers have developed multi-modal drying strategies based on real-time material moisture content by coupling infrared online monitoring with deep learning algorithms. This innovation not only enhances drying efficiency but also effectively prevents nutritional loss issues such as starch gelatinization through regulated intermittent microwave irradiation cycles. The construction of this intelligent drying system provides a novel technological pathway to overcome the energy efficiency limitations of traditional drying processes.

In recent years, extensive foundational research has focused on optimizing microwave drying parameters and modeling heat-mass transfer mechanisms. For instance, T. Sun et al. [17] investigated optimal conditions for improving microwave drying efficiency of rice. Utilizing orthogonal experimental principles, they analyzed factors influencing crack formation and whole-grain yield. By applying grey system theory, they proposed a parameter optimization method that simultaneously considers crack rate and whole-grain yield, conducting grey relational analysis on microwave drying parameters. Results indicated that vacuum level predominantly affects whole-grain yield, while loading thickness primarily influences crack rate, with significant fluctuations in grey correlation curves and high interdependency between the two metrics. Sun et al. [18] established a moisture content variation model and simulated microwave heating processes coupling electromagnetic and thermal-mass fields to elucidate moisture diffusion and migration mechanisms during drying, validated by experimental data under varying vacuum conditions.

However, although existing research has made phased progress in optimizing drying parameters and simulating heat and mass transfer, the focus has predominantly been on macroscopic drying efficiency and physical quality indicators (such as cracking rate and head rice yield) or has been limited to the investigation of single moisture migration mechanisms. A key scientific question that remains insufficiently systematically elucidated is: during the multi-field coupled microwave drying process, how do the multi-dimensional quality indicators within rice kernels (such as mineral elements, vitamins, and other nutritional components, as well as free fatty acid content indicative of aging rate) dynamically evolve in response to key process parameters, and what synergistic or antagonistic relationships exist among these quality indicators. Existing models struggle to accurately quantify the interactive effects of parameters such as microwave power, hot air temperature, and grain bed thickness on this complex quality system, and there is a lack of process control strategies aimed at synergistically optimizing multiple nutritional qualities and storage stability.

Therefore, to explore effective strategies and influence mechanisms for transitioning from macro-parameter optimization to micro-quality synergistic regulation, this study established a unified evaluation system encompassing four key indicators: potassium (K), calcium (Ca), vitamin B1 (VB1), and free fatty acids (FFA), to systematically investigate their dynamic evolution patterns during the combined microwave-hot air drying process. Based on a microwave grain dryer, the research employed a Box-Behnken design (microwave power: 10–20 kW; hot air temperature: 40–60°C; grain bed thickness: 1.5–3.5 cm) to systematically analyze the influence patterns and interaction mechanisms of key parameters on the multi-dimensional quality indicators of rice under multi-field coupled conditions. On this basis, a dynamic response model for drying quality was constructed, and process reference maps were established to reveal the mapping relationship between comprehensive quality evolution and drying parameter combinations, aiming to provide a theoretical basis for achieving high-quality microwave drying of rice.

## Materials and methods

### Experimental setup

**Fig 1:**
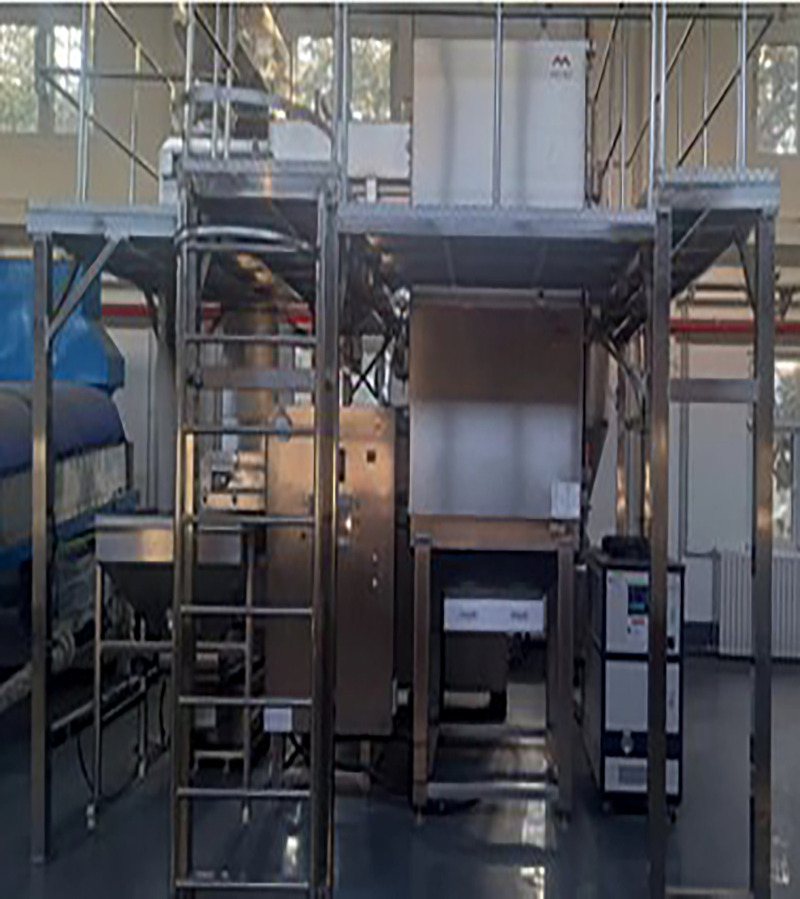
Microwave grain drying experimental apparatus.

The experiments were conducted using a microwave grain drying test machine developed by Jiangsu Micwave Microwave Technology Co., Ltd., as shown in Fig 1. The drying system primarily consisted of the following components: the main drying chamber, a transmission system, a microwave heating compartment (with adjustable microwave power ranging from 10 to 20 kW), a tempering chamber, a fan (equipped with a variable-frequency speed regulator, adjustable airflow velocity: 30–50 m/s), a heating unit (controllable hot air temperature range: 30–80°C), and a control system.Other experimental instruments and equipment are shown in Table 1.

**Table 1:** Test instruments and manufacturers.

Name of instrument and equipment	Manufacturing enterprise
DGG-9250GD Electric Thermostatic Blast Drying Oven	Shanghai Senxin Laboratory Instrument Co., Ltd
FZ102 Mini Plant Sample Grinder	Beijing Yongguangming Medical Instrument Co., Ltd.
PQ-520 Grain Moisture Meter	Shandong Yunling Intelligent Technology Co., Ltd
ES-S/B Electronic Balance	Hebei Xinteng Biotechnology Co., Ltd., accuracy: 0.01 g
BSA223S Analytical Balance	Sartorius, Germany, accuracy: 0.0001 g
GDH High-Precision Constant-Temperature Water Bath	Jiangsu Tianling Instrument Co., Ltd.
UV1700 UV-Vis Spectrophotometer	Shanghai Aoxi Scientific Instrument Co., Ltd.
Controlled Tube Grinder	IKA Works, Guangzhou, China
OS-200 Orbital Shaker	Hangzhou Aosheng Instrument Co., Ltd.
TG16K Centrifuge	Changsha Dongwang Laboratory Instrument Co., Ltd.
EVO18 Scanning Electron Microscope	Carl Zeiss AG, Germany

### Experimental materials

The tested paddy rice was the freshly harvested high-quality early indica rice variety Yongxian 634 (Ningbo, China), with an initial wet-basis moisture content of approximately 30%.

### Experimental methods

The raw materials were pre-processed to remove impurities and immature grains. All experiments were conducted in July 2024 at the Changping Pilot Base of the Academy of National Food and Strategic Reserves Administration.

To ensure the reliability and statistical significance of the experimental results, all designated combinations of hot air temperature, microwave power, and grain bed thickness were conducted in three independent replicates. Prior to each test group, the initial moisture content of the rice samples was re-measured, and system stability verification was performed. An intermittent drying strategy (10-min heating/ 30-min tempering) was adopted during the process, aiming to promote uniform internal moisture diffusion through periodic tempering stages while avoiding localized overheating issues caused by continuous heating, such as starch gelatinization, intensified Maillard reaction, or degradation of heat-sensitive components. On this basis, by parametrically controlling the combination of hot air temperature and microwave power, the dynamic changes in material temperature and moisture content were monitored in real-time to facilitate subsequent rapid processing of experimental data [19–20]. Drying was terminated when the moisture content reached the safe threshold range of 14.0%–14.5%, and the samples were transferred to a low-temperature environment for storage. Upon completion of each parameter combination test, fresh samples were replaced for the next replicate, and this procedure was repeated until all tests were concluded.

Ultimately, all analyses were based on the mean values derived from the three replicate experiments.

### Measurement of experimental indicators

#### Moisture content.

Measured using a JC-LS-01S halogen moisture analyzer.

#### Potassium and calcium content.

With reference to the second method of GB 5009.268−2016 “National Food Safety Standard—Determination of Multiple Elements in Foods,” the measurement is conducted as follows:Take the rice sample and grind it uniformly using a high-speed grinder. Accurately weigh 0.5 ~ 5 g (accurate to 0.001 g) of the sample into a glass or polytetrafluoroethylene digestion vessel. For samples containing ethanol or carbon dioxide, first remove the ethanol or carbon dioxide by heating on an electric hotplate at low temperature. Add 10 mL of a nitric acid–perchloric acid (10 + 1) mixture and digest on a hotplate or graphite digestion system. If the digestion solution turns brownish-black during the process, an appropriate amount of the mixed acid may be added as needed. Continue digestion until white fumes are emitted and the digest turns colorless, transparent, or slightly yellow. Allow the solution to cool, then dilute to a final volume of 25 mL or 50 mL with water, and mix well for subsequent use. A blank test is performed simultaneously.Accurately pipette an appropriate amount of potassium or calcium elemental standard stock solution and prepare a series of mixed standard solutions by stepwise dilution with nitric acid solution (5 + 95). Inject the standard series working solutions into an inductively coupled plasma optical emission spectrometer (ICP-OES) and measure the intensity signal response of the analytical spectral line for the target element. Plot the standard curve using the concentration of the target element as the abscissa and the intensity response of the analytical spectral line as the ordinate. Then, inject the blank solution and the sample solution separately into the ICP-OES, measure the intensity signal response of the analytical spectral line for the target element, and determine the concentration of the target element in the digest based on the standard curve.

Formula: $\text{X}=\frac{\left(\text{p}-\text{p0}\right)\times \text{V}\times \text{f}}{\text{m}}$, where:X is the content of the target element in the sample (mg/kg);p is the mass concentration of the target element in the sample solution (mg/L);p0 is the mass concentration of the target element in the sample blank solution (mg/L);V is the final volume of the sample digest (mL);f is the dilution factor of the sample;m is the mass of the sample weighed (g).

#### Vitamin B1 content.

With reference to the first method of GB 5009.84−2016 “National Food Safety Standard—Determination of Vitamin B₁ in Foods,” the measurement is conducted as follows:Weigh 3 ~ 5 g (accurate to 0.01 g) of rice sample into a 100 mL conical flask (fitted with a soft stopper). Add 60 mL of 0.1 mol/L hydrochloric acid solution, mix thoroughly, stopper with the soft plug, and maintain at 121°C in a high-pressure sterilizer for 30 minutes. After hydrolysis, allow the mixture to cool below 40°C before removal, and shake gently several times. Using a pH meter for indication, adjust the pH to around 4.0 with 2.0 mol/L sodium acetate solution. Add 2.0 mL of mixed enzyme solution, mix well, and incubate in a constant temperature incubator at 37°C overnight (approximately 16 hours). Transfer the entire enzymatically digested solution into a 100 mL volumetric flask, dilute to volume with water, mix well, and centrifuge or filter. Collect the supernatant for subsequent use.Next, accurately transfer 2.0 mL of the supernatant or filtrate into a 10 mL test tube. Add 1.0 mL of alkaline potassium ferricyanide solution, vortex to mix thoroughly, then accurately add 2.0 mL of n-butanol, and vortex again for 1.5 minutes. Allow the mixture to stand for about 10 minutes or centrifuge. After complete phase separation, draw the n-butanol phase and filter it through a 0.45 μm organic microporous membrane. Collect the filtrate in a 2 mL brown injection vial for analysis. If the concentration of vitamin B₁ in the test solution exceeds the upper limit of the linear range, an appropriate dilution of the supernatant should be performed before repeating the derivation and injection. Additionally, take 2.0 mL of the standard series working solution and carry out derivatization simultaneously with the test solution. Inject the derivatives of the standard series working solutions into a high-performance liquid chromatograph, measure the corresponding peak areas of vitamin B₁, and plot the standard curve with the concentration (μg/mL) of the standard working solutions as the abscissa and the peak area as the ordinate. Inject the derivative solution of the sample into the high-performance liquid chromatograph to obtain the peak area of vitamin B₁, and calculate the concentration of vitamin B₁ in the test solution based on the standard curve.

Formula:X$=\frac{\text{c}\times \text{V}\times \text{f}}{\text{m}\times \text{1000}}\times \text{100}$,where:X is the content of vitamin B₁ in the sample (mg/100 g);c is the concentration of vitamin B₁ in the test solution calculated from the standard curve (μg/mL);V is the final volume of the test solution (mL);f is the dilution factor of the test solution before derivatization;*mm* is the mass of the sample (g).

#### Free fatty acid content.

The determination shall be carried out with reference to GB/T 20569−2006 “Rules for Judgment of Paddy Storage Quality”. The specific steps are as follows: Weigh a uniformly mixed paddy sample and hull it using a laboratory husker. Take approximately 80g of the uniformly mixed brown rice and grind it with a hammer cyclone mill. Over 95% of the ground sample should pass through a CQ16 sieve in one go. After thorough mixing, transfer the ground sample into a ground-mouth bottle for later use. Then, weigh about 10g of the prepared sample, accurate to 0.01g, into a 250mL ground-glass stoppered conical flask. Add 50.0mL of anhydrous ethanol using a pipette, and shake the flask for 10 minutes on a reciprocating shaker at a frequency of 100 oscillations per minute. Allow the mixture to stand for 1 ~ 2 minutes, and filter it through a folded filter paper placed in a glass funnel. Discard the first few drops of the filtrate and collect more than 25mL of the filtrate. Transfer 25.0mL of the filtrate into a 150mL conical flask using a pipette, add 50mL of carbon dioxide-free distilled water, and then add 3 ~ 4 drops of phenolphthalein indicator. Titrate the mixture with a standard potassium hydroxide titration solution until a faint pink color appears and persists for 30 seconds. Record the volume of the standard potassium hydroxide titration solution consumed (V1). Transfer 25.0mL of anhydrous ethanol into a 150mL conical flask using a pipette, add 50mL of carbon dioxide-free distilled water, and then add 3 ~ 4 drops of phenolphthalein indicator. Titrate the mixture with a standard potassium hydroxide titration solution until a faint pink color appears and persists for 30 seconds. Record the volume of the standard potassium hydroxide titration solution consumed (V0).

Formula:S=(V1−V0)×c×56.1×2$\times \frac{\text{100}}{\text{m}(\text{100}-\text{w})}\times$100, Where:S is the free fatty acid content of the sample (mg/100g);V1 is the volume of standard potassium hydroxide titration solution consumed in titrating the sample (mL);V0 is the volume of standard potassium hydroxide titration solution consumed in titrating the blank solution (mL);c is the concentration of the standard potassium hydroxide titration solution (mol/L);m is the mass of the sample (g);w is the mass fraction of moisture in the sample (g).

### Process reference map

A process reference map was constructed based on a quadratic regression model [21–22]. Hot air temperature (T) and microwave power (P) were selected as key control variables, with their ranges defined as the axes (T: 40–60°C; P: 10–20 kW). Contour plots were generated by superimposing constraints (e.g., potassium K, calcium Ca, vitamin B1 VB1, and free fatty acid retention FFA) and correlating them with grain layer thicknesses (h) (1.5 cm, 2.5 cm, 3.5 cm), thereby establishing a multi-objective collaborative optimization framework.

Note: T denotes hot air temperature; P denotes microwave power; h denotes grain layer thickness. K denotes potassium content; Ca denotes calcium content; VitB1 denotes vitamin B1 content; FFA denotes free fatty acid content.

### Orthogonal experimental design

Using hot air temperature, microwave power, and grain layer thickness as experimental factors, and potassium (K), calcium (Ca), vitamin B1 (VB1), and free fatty acid (FFA) content as experimental indicators, a Box-Behnken design [23] was employed to investigate the interactive effects of the factors on the drying indicators, thereby optimizing the parameters for the homogeneous drying process of paddy rice. The coding table for the orthogonal tests is shown in Table 2.

**Table 2:** Coding of orthogonal experimental factors.

Coding	Factor		
	Hot air temperature/(°C)	Microwave power/(kW)	Grain layer thickness/(cm)
−1	40	10	1.5
0	50	15	2.5
1	60	20	3.5

### Data processing

Experimental data were processed and analyzed using Microsoft Excel. The Box-Behnken experimental design was generated using the Box-Behnken module in Design-Expert® software (Version 13). Statistical analysis and graphical plotting were performed using OriginPro 2021 software.

## Results and analysis

### Analysis of orthogonal experimental results

#### Experimental design and outcomes.

The effects of various factors on response variables and their interactions were investigated through experimentation. The experimental design [24] and corresponding results are summarized in Table 3.

**Table 3:** Orthogonal experimental design and results of microwave drying process for rice.

Experiment No.	T(°C)	P(kW)	H(cm)	K(mg/kg)	Ca(mg/kg)	W(mg/100g)	FFA(mg/100g)
1	40	10	2.5	3770	118.0	0.269	34.1
2	40	20	2.5	3650	114.0	0.253	22.1
3	60	10	2.5	3300	93.1	0.282	26.1
4	60	20	2.5	3620	107.0	0.301	22.3
5	50	10	1.5	3590	106.0	0.269	22.6
6	50	20	1.5	3360	116.0	0.286	11.1
7	50	10	3.5	3470	108.0	0.287	26.1
8	50	20	3.5	3720	107.0	0.285	20.4
9	40	15	1.5	3550	113.0	0.236	20.4
10	60	15	1.5	3240	87.7	0.294	19.0
11	40	15	3.5	3570	108.0	0.280	26.9
12	60	15	3.5	3420	98.4	0.278	26.5
13	50	15	2.5	3660	114.0	0.286	24.9
14	50	15	2.5	3600	109.0	0.278	25.5
15	50	15	2.5	3690	112.0	0.279	26.3

Note: Experiments 13, 14, and 15 are replicate runs at the center point to estimate experimental error.

#### Regression model fitting and ANOVA for microwave drying of paddy rice.

Based on the experimental data, the study established second-order polynomial nonlinear regression models for potassium content, calcium content, vitamin B1 content, and free fatty acid content, respectively, using Design-Expert software. The final regression equations, obtained after eliminating non-significant factors, are shown in Table 4. The coefficient of determination (R²) for each model was greater than 0.96, indicating that the models explain over 96% of the response variability, demonstrating a good goodness-of-fit.To facilitate the assessment of the relative influence strength of each factor, Table 4 also presents the regression equations in coded units. Here, the independent variables A (air temperature), B (microwave power), and C (grain bed thickness) have been coded, with their low and high levels corresponding to −1 and +1, respectively. In the coded equations, the absolute value of a coefficient reflects the strength of the factor’s influence, while the sign of the coefficient indicates the direction of the effect.Furthermore, the analysis of variance (ANOVA) results for the regression models of each response variable are presented in Tables 5–8.

**Table 4:** Second-order polynomial nonlinear regression model equations.

Indicator	Model type	Second-order polynomial model equations	R^2^
K	Coded Equation	Z = 3650−120 × A + 27.5 × B + 55 × C + 110 × A*B + 40 × A*C + 120 × B*C-77.5 × A^2^ + 12.5 × B^2^-127.5 × C^2^	0.975
	Actual Equation	Z = 4458.125 + 22.5 × T-179.5 × P + 132.5 × h + 2.2 × T*P + 4 × T*h + 24 × P*h-0.775 × T^2^ + 0.5 × P^2^-127.5 × h^2^	
Ca	Coded Equation	Z = 111.67–8.35 × A + 2.36 × B-0.1625 × C + 4.48 × A*B + 3.92 × A*C-2.75 × B*C-5.56 × A^2^ + 1.92 × B^2^-4.33 × C^2^	0.9661
	Actual Equation	Z = 93.50625 + 2.39958 × T-4.9275 × P + 10.12917 × h + 0.0895 × T*P + 0.3925 × T*h-0.55 × P*h-0.055583 × T^2^ + 0.076667 × P^2^-4.33333 × h^2^	
VitB1	Coded Equation	Z = 0.281 + 0.0146 × A + 0.0022 × B + 0.0056 × C + 0.0088 × A*B-0.015 × A*C-0.0047 × B*C-0.0072 × A^2^ + 0.0025 × B^2^-0.0018 × C^2^	0.9704
	Actual Equation	Z = −0.0745 + 9.837 × 10^-3^T-8.925 × 10^-3^P + 0.103625 × h + 1.75 × 10^-4^T*P-1.5 × 10^-3^T*h-9.5 × 10^-4^P*h-7.2 × 10^-5^T^2^+10^-4^P^2^-1.75 × 10^-3^h^2^	
FFA	Coded Equation	Z = 25.57–1.2 × A-4.13 × B + 3.35 × C + 2.05 × A*B + 0.25 × A*C + 1.45 × B*C + 1.87 × A^2^-1.28 × B^2^-4.23 × C^2^	0.9835
	Actual Equation	Z = 88.975-2.66417 × T-2.06 × P + 18.91667 × h + 0.041 × T*P + 0.025 × T*h + 0.29 × P*h + 0.018667 × T^2^-0.051333 × P^2^-4.23333 × h^2^	

Note: A is the hot-air temperature, B is the microwave power, and C is the grain layer thickness.

**Table 5:** Analysis of variance (ANOVA) for the potassium content regression model.

Source	Sum of squares	Degrees of freedom	Mean square	F value	P	Remark
model	3.376 × 10^5^	9	37515.93	21.69	0.0017	significant
A-T	1.152 × 10^5^	1	1.152 × 10^5^	66.59	0.0004	
B-P	6050	1	6050	3.5	0.1204	
C-h	24200	1	24200	13.99	0.0134	
A*B	48400	1	48400	27.98	0.0032	
A*C	6400	1	6400	3.7	0.1124	
B*C	57600	1	57600	33.29	0.0022	
A^2^	22176.92	1	22176.92	12.82	0.0159	
B^2^	576.92	1	576.92	0.3335	0.5886	
C^2^	60023.08	1	60023.08	34.7	0.002	
residual	8650	5	1730			
Misspecification term	4450	3	1483.33	0.7063	0.631	insignificant
Pure error	4200	2	2100			
sum	3.463 × 10^5^	14				

**Table 6:** Analysis of variance (ANOVA) for the vitamin B1 content regression model.

Source	Sum of squares	Degrees of freedom	Mean square	F value	P	Remark
model	0.0035	9	0.0004	26.47	0.0011	significant
A-T	0.0017	1	0.0017	115.23	0.0001	
B-P	0	1	0	2.73	0.1596	
C-h	0.0003	1	0.0003	17.05	0.0091	
A*B	0.0003	1	0.0003	20.62	0.0062	
A*C	0.0009	1	0.0009	60.61	0.0006	
B*C	0.0001	1	0.0001	6.08	0.0569	
A^2^	0.0002	1	0.0002	13.07	0.0153	
B^2^	0	1	0	1.55	0.2678	
C^2^	0	1	0	0.7615	0.4228	
residual	0.0001	5	0			
Misspecification term	0	3	0	0.636	0.6589	insignificant
Pure error	0	2	0			
sum	0.0036	14				

**Table 7:** Analysis of variance (ANOVA) for the calcium content regression model.

Source	Sum of squares	Degrees of freedom	Mean square	F value	P	Remark
model	971.04	9	107.89	15.84	0.0036	significant
A-T	557.78	1	557.78	81.9	0.0003	
B-P	44.65	1	44.65	6.56	0.0506	
C-h	0.2112	1	0.2112	0.031	0.8671	
A*B	80.1	1	80.1	11.76	0.0186	
A*C	61.62	1	61.62	9.05	0.0298	
B*C	30.25	1	30.25	4.44	0.0889	
A^2^	114.07	1	114.07	16.75	0.0094	
B^2^	13.56	1	13.56	1.99	0.2173	
C^2^	69.33	1	69.33	10.18	0.0242	
residual	34.05	5	6.81			
Misspecification term	21.39	3	7.13	1.13	0.5023	insignificant
Pure error	12.67	2	6.33			
sum		14				

**Table 8:** Analysis of variance (ANOVA) for the free fatty acid content regression model.

Source	Sum of squares	Degrees of freedom	Mean square	F value	P	Remark
model	351.55	9	39.06	33.04	0.0006	significant
A-T	11.52	1	11.52	9.74	0.0262	
B-P	136.13	1	136.13	115.13	0.0001	
C-h	89.78	1	89.78	75.93	0.0003	
A*B	16.81	1	16.81	14.22	0.013	
A*C	0.25	1	0.25	0.2114	0.6649	
B*C	8.41	1	8.41	7.11	0.0445	
A^2^	12.87	1	12.87	10.88	0.0215	
B^2^	6.08	1	6.08	5.14	0.0726	
C^2^	66.17	1	66.17	55.97	0.0007	
residual	5.91	5	1.18			
Misspecification term	4.93	3	1.64	3.33	0.2396	insignificant
Pure error	0.9867	2	0.4933			
sum	357.46	14				

### Analysis of multi-factor interaction effects on potassium content variation

The analysis of variance results for the potassium content regression model are presented in Table 5. The model was highly significant (P = 0.0017 < 0.01), explaining 97.5% of the variation in paddy rice potassium content (R² = 0.975). The non-significant lack-of-fit term (P = 0.631 > 0.05) indicates a good model fit. Based on the absolute values of the linear term coefficients in the coded equation (Z = 3650 − 120 × A + 27.5 × B + 55 × C + 110 × A*B + 40 × A*C + 120 × B*C − 77.5 × A² + 12.5 × B² − 127.5 × C²), the primary and secondary order of factors affecting potassium content and their effect directions were determined. The order of influence was: Hot-air temperature (A)> Grain layer thickness (C)> Microwave power (B).Hot-air temperature (A) was the most influential factor, exhibiting a significant negative effect (coefficient = −120, contribution rate 34.1%). This result aligns with the conclusion of Cao et al. [25] that temperature dominates mineral loss. Our study further quantifies the specific contribution rate of hot-air temperature within this complex coupled drying model, providing data support for its dominant role. The mechanism lies in the fact that high temperature induces the denaturation and inactivation of potassium ion channel proteins, while simultaneously increasing the kinetic energy of phospholipid molecules in the cell membrane. This triggers a transition of the membrane structure from an ordered gel state to a disordered liquid crystalline state, weakening its selective barrier function and thereby accelerating the diffusion and leaching of potassium ions.Grain layer thickness (C) was a key positive effect factor (coefficient=+55, contribution rate 28.5%). Contrary to the general understanding in pure hot-air drying where thick layers exacerbate component degradation due to high heat and mass transfer resistance, in the microwave-coupled field, a moderate grain layer thickness might modulate the spatial distribution of microwave energy, influence the spatiotemporal heterogeneity of the internal temperature and moisture fields, and consequently form a micro-environment favorable for potassium retention. Furthermore, its significant negative quadratic term (C² = −127.5) indicates a parabolic relationship between potassium content and layer thickness, suggesting the existence of a specific thickness range that maximizes potassium content, which should be considered in practical operation.The independent effect of microwave power (B) did not reach significance (P = 0.1204), but its positive coefficient (+27.5) and significant interaction effects indicate that its mechanism of action in the coupled drying system is complex and its effect is highly dependent on other parameters. This explains the divergent conclusions regarding its dominance across different research systems.

**Fig 2:**
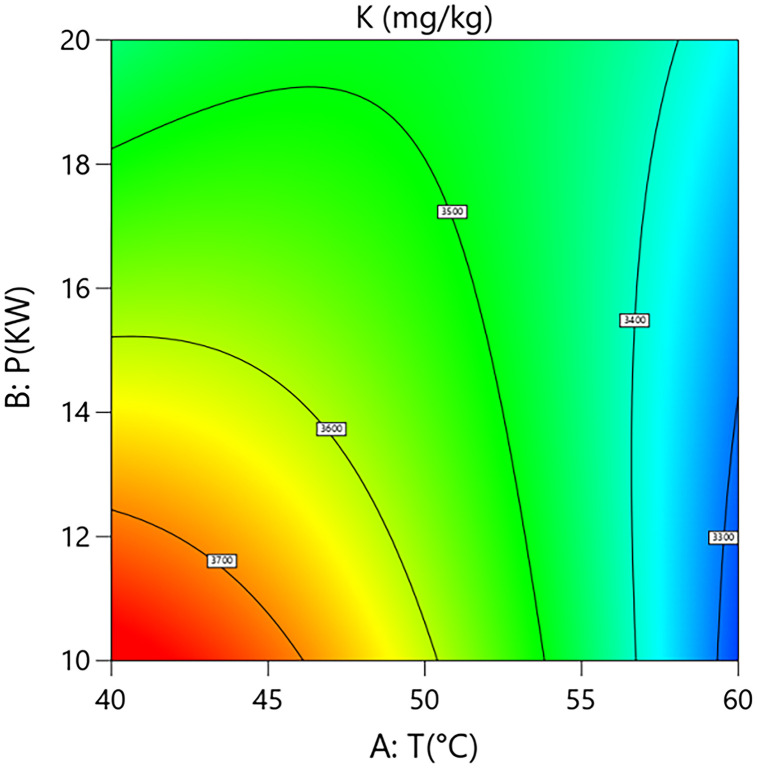
Effect of the interaction between hot air temperature (T) and Microwave Power (P) on Potassium Content in Rice:Contour Plot.

**Fig 3:**
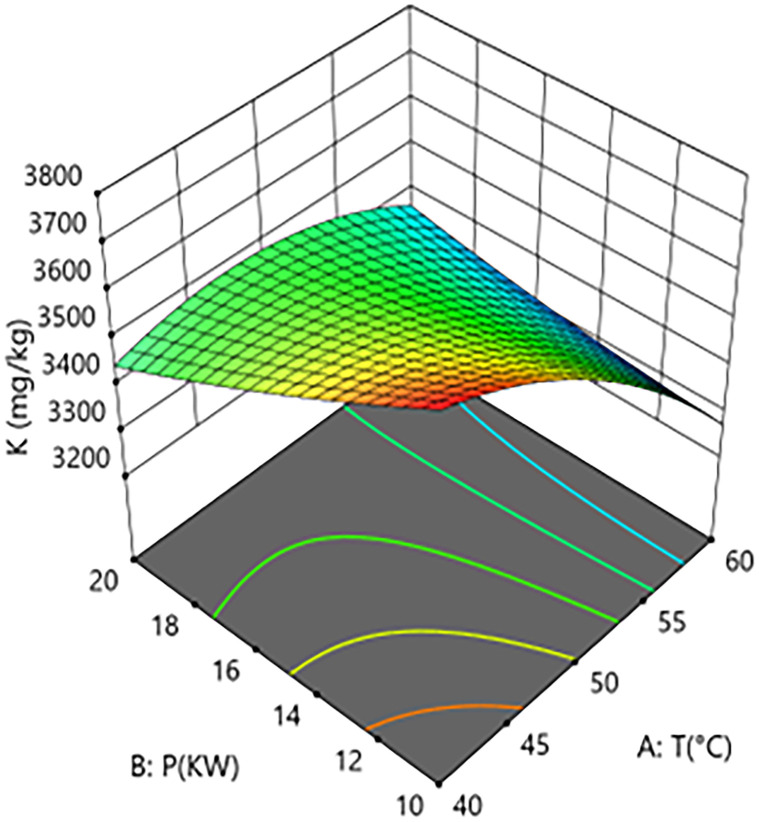
Effect of the interaction between hot air temperature (T) and Microwave Power (P) on Potassium Content in Rice:Response Surface Plot.

**Fig 4:**
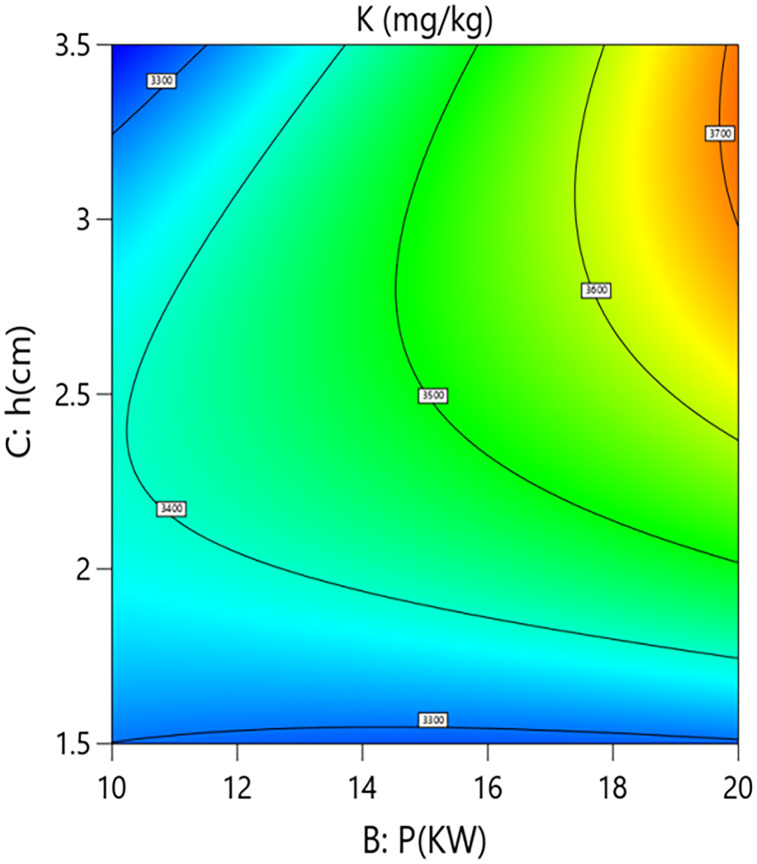
Effect of the interaction between microwave power (P) and Grain Layer Thickness (h) on Potassium Content in Rice: Contour Plot.

**Fig 5:**
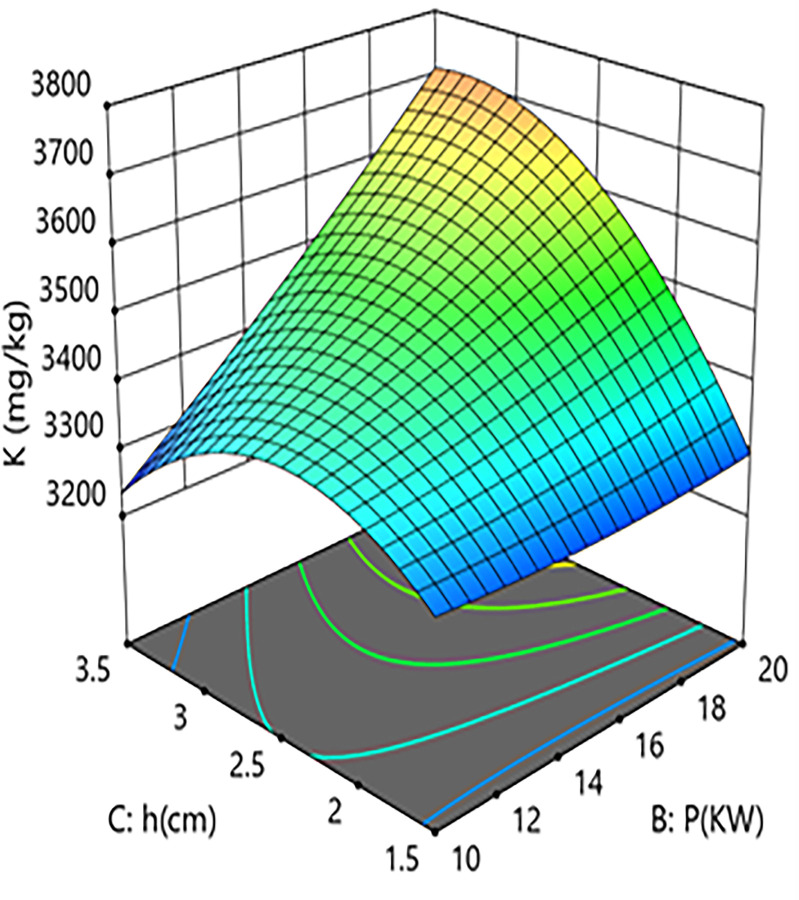
Effect of the interaction between microwave power (P) and Grain Layer Thickness (h) on Potassium Content in Rice:Response Surface Plot.

Interaction analysis further revealed the complex coupling mechanisms between the parameters. As shown in Figs 2 and 3, a significant positive synergistic effect exists between hot-air temperature and microwave power (A × B coefficient=+110), indicating that an increase in microwave power exacerbates the negative impact of hot-air temperature on potassium loss. Response surface analysis showed that with grain layer thickness fixed at the center point, when process conditions shifted from “low temperature–low power” (40°C, 10 kW) to “high temperature–high power” (60°C, 20 kW), the potassium content decreased significantly from 3680 mg/kg to 3320 mg/kg, an absolute reduction of 360 mg/kg (relative decrease of 9.8%). This demonstrates that the combination of high temperature and high power has an additive effect, causing more internal regions of the material to rapidly exceed the membrane phase transition critical point, thereby intensifying potassium loss.As shown in Figs 4 and 5, a strong positive interaction exists between microwave power and grain layer thickness (B × C coefficient=+120), indicating that increasing grain layer thickness enhances the positive influence of microwave power on potassium content. Response surface analysis quantitatively revealed this non-linear relationship: under thin layer conditions (1.5 cm), increasing power from 10 kW to 20 kW resulted in a 6.4% decrease in potassium content (from 3590 mg/kg to 3360 mg/kg). In contrast, under thick layer conditions (3.5 cm), the same power increase led to a 7.2% rise in potassium content (from 3470 mg/kg to 3720 mg/kg). This phenomenon highlights the complexity of the coupled drying system: in thick-layer materials, although the skin effect of microwaves may cause energy concentration at the surface, appropriately increasing power might enhance internal steam pressure-driven moisture migration or improve hot-air convection efficiency, optimizing the overall drying kinetics and consequently benefiting potassium retention in net effect. This finding goes beyond the observations of Deng et al. [26] who focused solely on uneven heat distribution, by revealing a more complex interaction mechanism between energy input and material structure.

In summary, through quantitative modeling and mechanistic analysis, the study clarified the core negative effect of hot-air temperature on potassium loss and revealed the dual role of microwave power in the coupled drying system: on one hand, it synergizes with high temperature to exacerbate potassium loss; on the other hand, it synergizes with grain layer thickness under thick-layer conditions to promote potassium retention. Therefore, to reduce potassium loss, process optimization should avoid the “high temperature–high power” combination. When processing thick-layer materials, employing medium to high microwave power can be considered to leverage its positive synergistic effect.

### Analysis of multi-factor interaction effects on Vitamin B1 content variation

The analysis of variance results for the vitamin B1 content regression model are presented in Table 6. The model was highly significant overall (P = 0.0011 < 0.01), explaining 97.2% of the variation in vitamin B1 content (R² = 0.972), indicating a good fit. Based on the absolute values of the linear term coefficients in the coded equation (Z = 0.281 + 0.0146 × A + 0.0022 × B + 0.0056 × C + 0.0088 × A*B – 0.015 × A*C – 0.0047 × B*C – 0.0072 × A² + 0.0025 × B² – 0.0018 × C²), the primary and secondary order of factors affecting vitamin B1 retention was determined as: hot-air temperature (A)> grain layer thickness (C)> microwave power (B).Hot-air temperature (A) was the most influential factor, exhibiting a significant positive effect (coefficient +0.0146). This net effect appears divergent from expectations based on classical thermal degradation theory. It potentially stems from the significantly accelerated drying process in the microwave-hot air coupled drying system, where a temperature rise shortens the overall exposure time of vitamin B1 to the hygrothermal environment. The resulting “time-reduction effect” outweighs the promoting effect of “temperature increase” on the instantaneous degradation rate in the net outcome. This result coexists mechanistically with the conclusion of Yang et al. [27] that temperature is a primary driver of vitamin degradation, but our model further reveals that under coupled drying conditions, the “time-temperature trade-off effect” may dominate. This suggests a difference in the dominant mechanism of temperature’s role between pure hot-air drying and coupled drying systems; the former is governed primarily by chemical kinetics, while the latter is significantly regulated by physical heat and mass transfer processes. Furthermore, the significant negative quadratic term for hot-air temperature (A² = –0.0072) provides evidence for this trade-off, clearly defining an optimal process window beyond which the negative effects of thermal degradation increase non-linearly.Grain layer thickness (C) demonstrated a secondary positive effect (coefficient +0.0056, contribution rate 8.6%). This suggests that within the coupled system, a moderate grain layer thickness might create a more buffered micro-environment, avoiding localized extreme overheating, thereby exerting a certain positive influence on the overall retention of vitamin B1.

**Fig 6:**
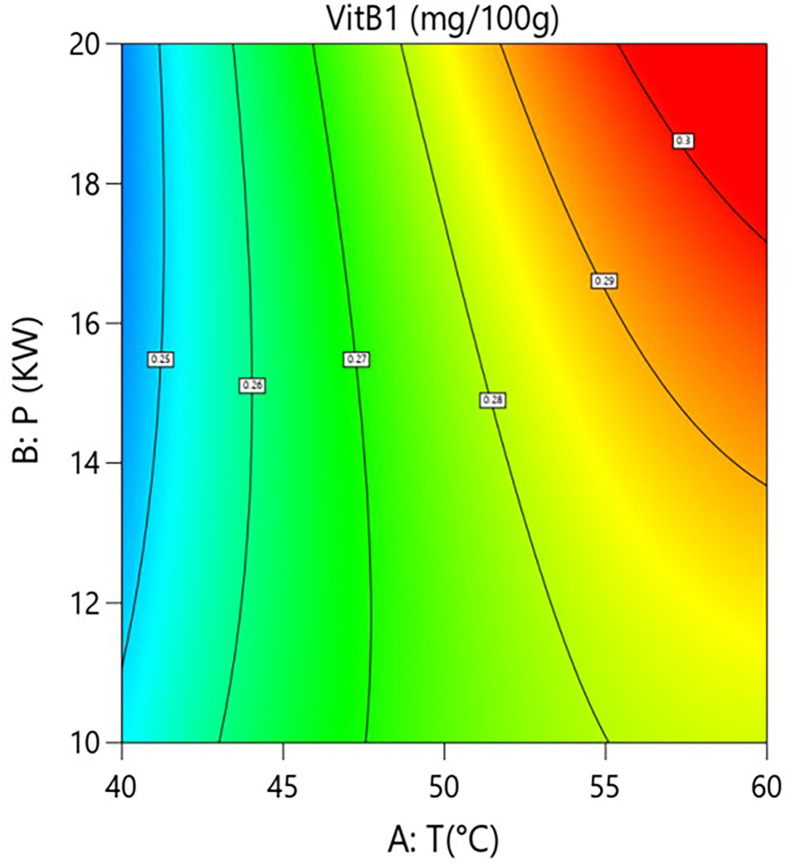
Effect of the interaction between hot air Temperature (T) and Microwave Power (P) on Vitamin B1 Content in Rice:Contour Plot.

**Fig 7:**
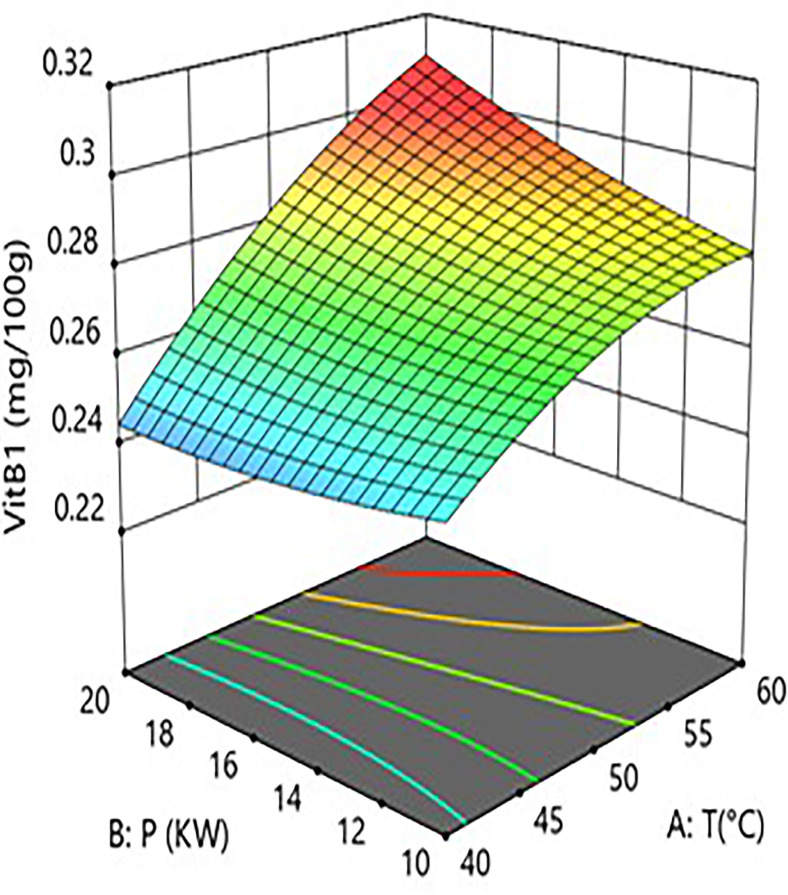
Effect of the interaction between hot air Temperature (T) and Microwave Power (P) on Vitamin B1 Content in Rice:Response Surface Plot.

**Fig 8:**
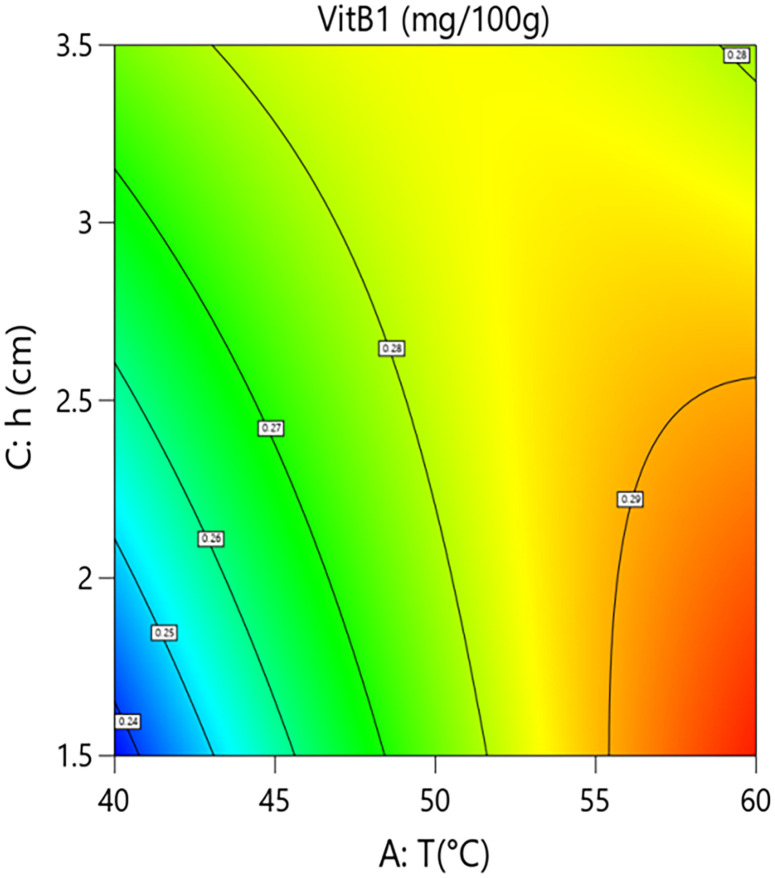
Effect of the interaction between hot air Temperature (T) and Grain Layer Thickness (h) on Vitamin B1 Content in Rice:Contour Plot.

**Fig 9:**
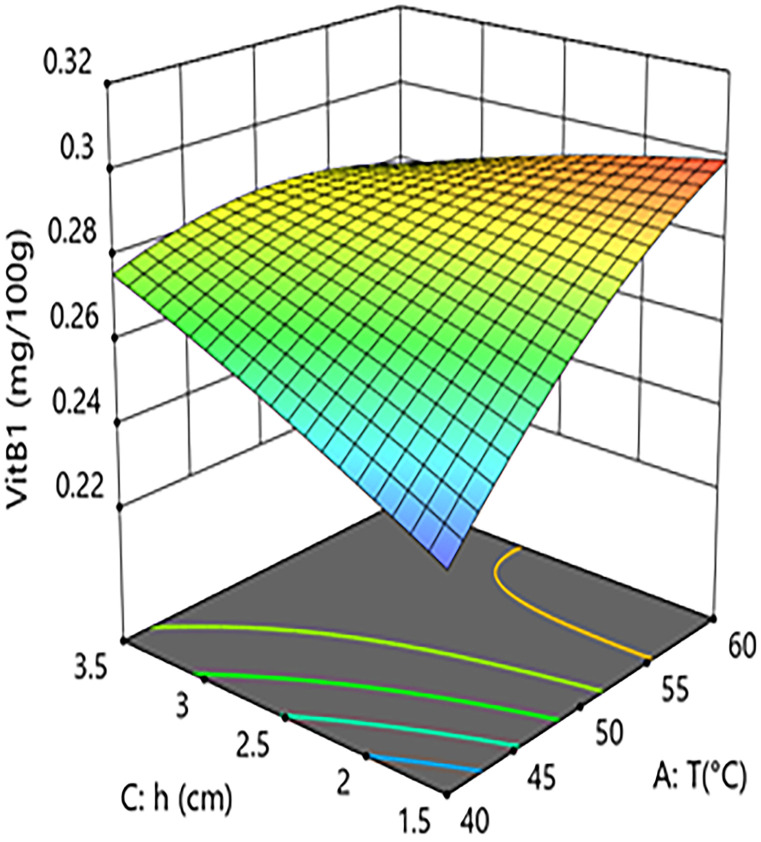
Effect of the interaction between hot air Temperature (T) and Grain Layer Thickness (h) on Vitamin B1 Content in Rice: Response Surface Plot.

Interaction analysis further revealed complex regulatory mechanisms between the factors. As shown in Figs 6 and 7, the interaction term between hot-air temperature and microwave power (A × B=+0.0088) was significant (P = 0.0062). This indicates that, within a specific parameter range, increasing microwave power enhances the positive effect of hot-air temperature on vitamin B1 retention. However, response surface analysis showed that when process parameters shifted from “low temperature–low power” (40°C, 10 kW) to “high temperature–high power” (60°C, 20 kW), the model-predicted vitamin B1 content decreased from approximately 0.285 mg/100g to 0.265 mg/100g, an absolute reduction of 0.020 mg/100g and a relative decrease of 7.0%. This indicates that despite the positive interaction, the independent negative trend of the hot-air temperature main effect is strong in parts of the range, resulting in its synergy with microwave power being insufficient to completely reverse the overall negative impact of high temperature in this specific transition.As shown in Figs 8 and 9, the interaction term between hot-air temperature and grain layer thickness (A × C = –0.015) was a key negative interaction term in the model. This indicates that increasing grain layer thickness significantly exacerbates the degradation effect of hot-air temperature on vitamin B1. Quantitative response surface analysis revealed that under high-temperature conditions (60°C), increasing the grain layer thickness from 1.5 cm to 3.5 cm caused the vitamin B1 content to drop from 0.294 mg/100g to 0.278 mg/100g, an absolute reduction of 0.016 mg/100g and a relative decrease of 5.4%. In contrast, at a lower temperature (40°C), the same change in thickness only resulted in a slight decrease in content from 0.285 mg/100g to 0.280 mg/100g, a reduction of 1.8%. This demonstrates that high temperature significantly amplifies the negative effect of a thick grain layer. The mechanism lies in the fact that a thick grain layer, under high-temperature conditions, impedes heat and mass transfer, leading to the accumulation of internal heat and moisture, thereby forming localized high-temperature and high-humidity “synergistic degradation zones” that drastically accelerate the degradation of vitamin B1. This phenomenon aligns with the results of Moraes [28] who observed gradient degradation of heat-sensitive components in thick grain layers during grain drying.

In summary, within the microwave-hot air coupled drying system, hot-air temperature exhibits a complex non-linear influence on vitamin B1 retention: within an optimized parameter range, it dominates a positive net effect by reducing drying time; however, in synergy with thick grain layers, it can trigger intensified localized degradation. Therefore, to maximize vitamin B1 retention, process optimization should adhere to the core principle of “moderate temperature and thin layer,” prioritizing a moderate hot-air temperature identified through quadratic model optimization and strictly controlling the grain layer thickness to avoid the risk of localized overheating caused by the synergy between high temperature and thick layers.

### Analysis of multi-factor interaction effects on calcium content variation

The analysis of variance results for the calcium content regression model are presented in Table 7. The quadratic model was highly significant (P = 0.0036 < 0.01), explaining 96.6% of the variation in calcium content (R² = 0.966). Based on the absolute values of the linear term coefficients in the coded equation (Z = 111.67–8.35 × A + 2.36 × B – 0.1625 × C + 4.48 × A*B + 3.92 × A*C – 2.75 × B*C – 5.56 × A² + 1.92 × B² - 4.33 × C²), the primary and secondary order of factors affecting calcium content was determined as: hot-air temperature (A)> microwave power (B)> grain layer thickness (C).Hot-air temperature (A) was the most influential negative effect factor (coefficient −8.35). This result aligns with the conclusion of Dukare et al. [29] that hot-air temperature is a critical constraint on calcium retention. However, this study further reveals that within the microwave-hot air coupled field, its negative effect primarily operates through two counteracting mechanisms: firstly, high temperature rapidly inactivates endogenous phytase, inhibiting its hydrolysis of calcium phytate and thus reducing the generation of soluble calcium ions; secondly, high temperature exacerbates cellular structure disruption, promoting the substantial leaching of originally bound calcium ions and their loss via moisture migration. The final negative coefficient of factor A in the model indicates that the latter leaching effect significantly outweighs the potential fixation effect of the former, leading to a marked decrease in calcium content. Furthermore, its significant negative quadratic term (A² = −5.56) indicates a non-linear, accelerating trend of calcium loss with increasing temperature.Conversely, microwave power (B) exhibited a definite positive effect (coefficient +2.36). Its protective role stems from its unique rapid volumetric heating characteristic, which enables the material interior to very quickly traverse the optimal activity temperature range of phytase. This achieves targeted inactivation of phytase before cellular structure suffers severe damage from prolonged heat exposure, thereby retaining more calcium within the phytate and preventing subsequent leaching loss. This result is consistent in direction with the observations of Hao et al. [30] in a pure microwave drying system, while our coupled model further quantifies it as a definite positive contribution and elucidates that the core mechanism lies in rapidly bypassing the optimal activity temperature range of phytase, achieving “targeted inactivation”.The independent linear effect of grain layer thickness (C) was weak (coefficient −0.1625), with its influence manifested mainly through interaction terms.

**Fig 10:**
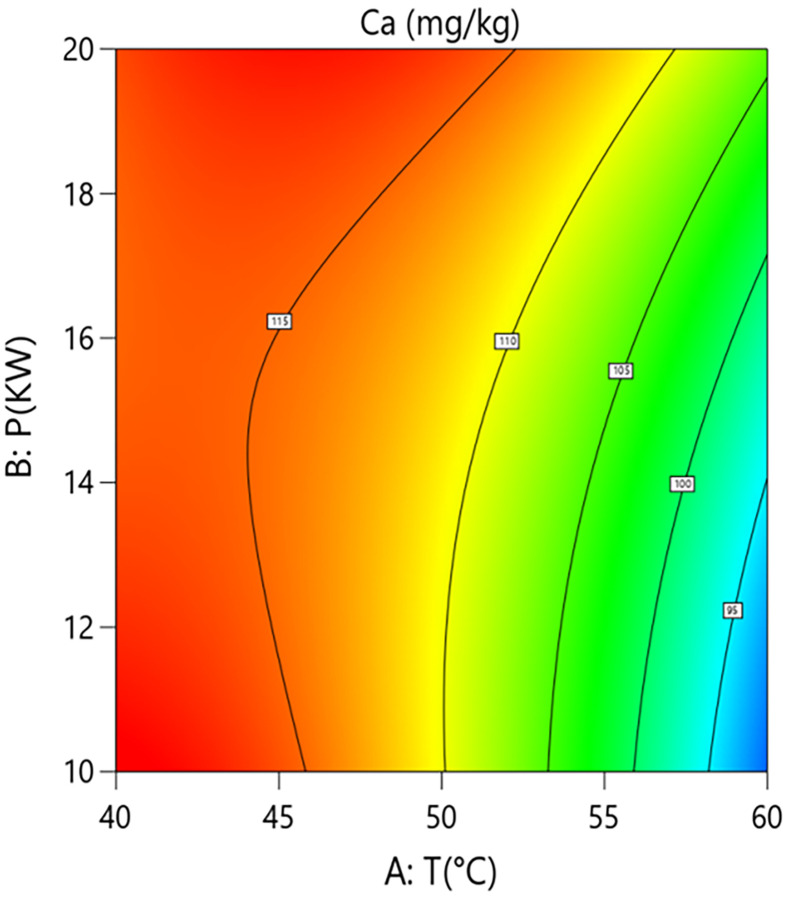
Effect of the interaction between hot air Temperature (T) and Microwave Power (P) on Calcium Content in Rice:Contour Plot.

**Fig 11:**
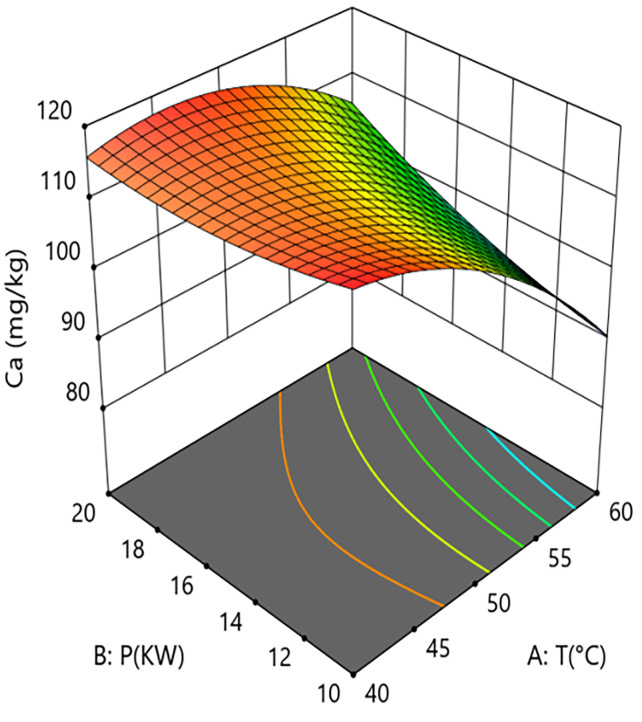
Effect of the interaction between hot air Temperature (T) and Microwave Power (P) on Calcium Content in Rice: Response Surface Plot.

**Fig 12:**
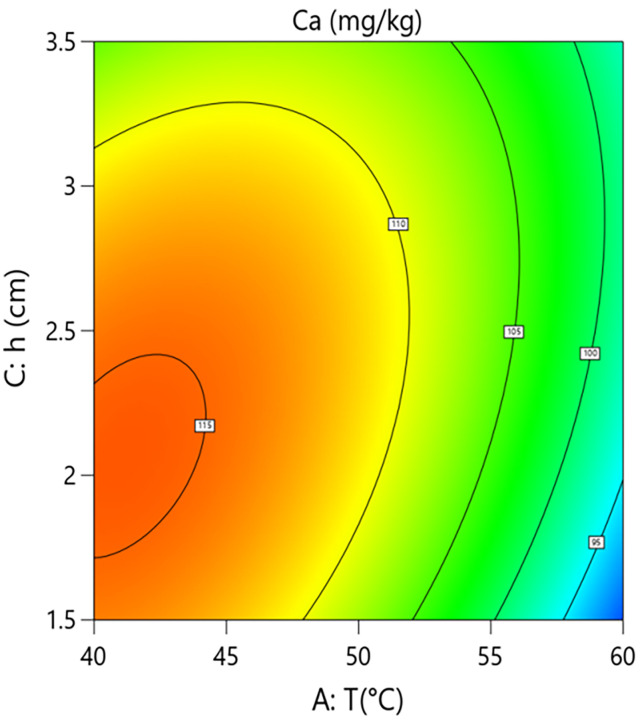
Effect of the interaction between hot air Temperature (T) and Grain Layer Thickness (h) on Calcium Content in Rice:Contour Plot.

**Fig 13:**
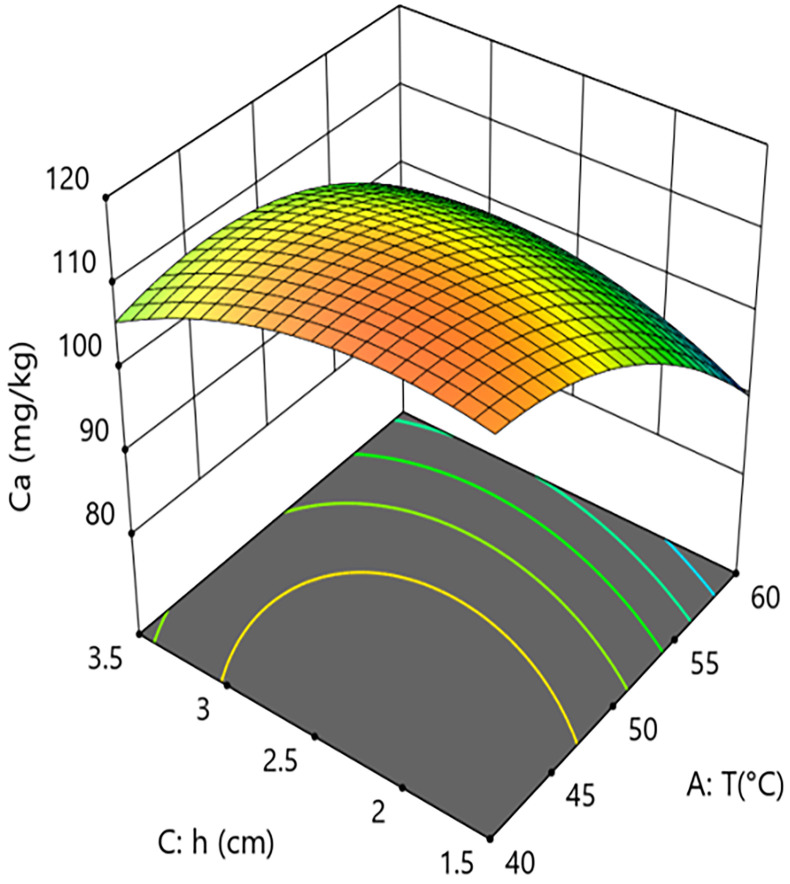
Effect of the interaction between hot air Temperature (T) and Grain Layer Thickness (h) on Calcium Content in Rice:Response Surface Plot.

Interaction analysis further revealed complex regulatory relationships between the parameters. As shown in Figs 10 and 11, the interaction term between hot-air temperature and microwave power (A × B=+4.48) had a significant effect on calcium content (P = 0.0186). This positive interaction coefficient indicates that increasing microwave power can effectively alleviate the calcium loss caused by rising hot-air temperature. Response surface analysis showed that at the medium grain layer thickness, when the system operated at low microwave power (10 kW), increasing the hot-air temperature from 40°C to 60°C caused calcium content to decrease from 118.0 mg/kg to 93.1 mg/kg, an absolute reduction of 24.9 mg/kg and a relative decrease of 21.1%. In contrast, under high microwave power (20 kW) conditions, the same temperature increase only led to a decrease in calcium content from 114.0 mg/kg to 107.0 mg/kg, a reduction of 6.1%. This result confirms the active role of microwaves in antagonizing the negative effect of high temperature within the coupled system, aligning with the conclusion of Li et al. (2022) that microwaves can compensate for the negative effects of hot air, while our study further clarifies the degree of mitigation via the interaction term coefficient.As shown in Figs 12 and 13, the interaction term between hot-air temperature and grain layer thickness (A × C = +3.92) was also significant (P = 0.0298). This positive interaction indicates that increasing grain layer thickness weakens the negative effect of hot-air temperature. Response surface analysis revealed that under high-temperature conditions (60°C), increasing the grain layer thickness from 1.5 cm to 3.5 cm raised the calcium content from 87.7 mg/kg to 98.4 mg/kg, an absolute increase of 10.7 mg/kg and a relative increase of 12.2%. Conversely, under low-temperature conditions (40°C), the same change in thickness only caused calcium content to decrease from 113.0 mg/kg to 108.0 mg/kg, a reduction of 4.4%. This phenomenon is attributed to the low-temperature “cold core” effect formed in the central region of thick grain layers, which provides a localized buffering micro-environment for calcium retention, thereby mitigating overall thermal damage.

In summary, calcium retention effectiveness is the result of a dynamic balance between the degradation effect dominated by hot-air temperature and the mitigation mechanisms constituted collectively by microwave power and grain layer thickness. To maximize the retention of calcium content in the material, the process should primarily involve strict control of hot-air temperature (recommended 50–55°C) to avoid its strongly non-linear negative effect. Secondly, medium to high microwave power (15–20 kW) should be employed to fully utilize its independent protective effect and its ability to antagonize the negative impact of high temperature. Finally, grain layer thickness should be judiciously controlled (2.0–3.0 cm) to ensure drying efficiency while leveraging the “cold core” effect it forms to provide spatial buffering for calcium retention.

### Analysis of multi-factor interaction effects on free fatty acid (FFA) content variation

Analysis of variance results for the free fatty acid (FFA) content regression model are presented in Table 8.The quadratic model was highly significant overall (P = 0.0006 < 0.01), explaining 98.3% of the variation in FFA content (R² = 0.983), indicating a good fit. Based on the absolute values of the linear term coefficients in the coded equation (Z = 25.57–1.20 × A – 4.13 × B + 3.35 × C + 2.05 × A*B + 0.25 × A*C + 1.45 × B*C + 1.87 × A² – 1.28 × B² – 4.23 × C²), the primary and secondary order of factors affecting FFA content was determined as: microwave power (B)> grain layer thickness (C)> hot-air temperature (A).Microwave power (B) was the most influential negative effect factor (coefficient −4.13, contribution rate 38.7%), indicating that increasing microwave power can effectively suppress FFA formation. This mechanism primarily stems from the rapid volumetric heating effect of microwaves, which can raise the temperature of the entire material mass in a very short time [31], thereby rapidly inactivating the key enzyme responsible for lipid hydrolysis—lipase. This result is consistent with the conclusion of Hao et al. [32] that microwave energy can inhibit lipid hydrolysis, but this study further quantified its dominant inhibitory role in the coupled drying system via modeling.Grain layer thickness (C) was the primary positive effect factor (coefficient +3.35, contribution rate 25.5%). Its significant negative quadratic term (C² = –4.23) indicated a parabolic relationship between FFA content and layer thickness, suggesting the existence of a specific thickness point that maximizes FFA content. This specific thickness region should be avoided in practice to control FFA accumulation. Hot-air temperature (A) was a secondary negative effect factor (coefficient –1.20). While its independent effect was relatively weak, it played a significant role in interaction effects.

**Fig 14:**
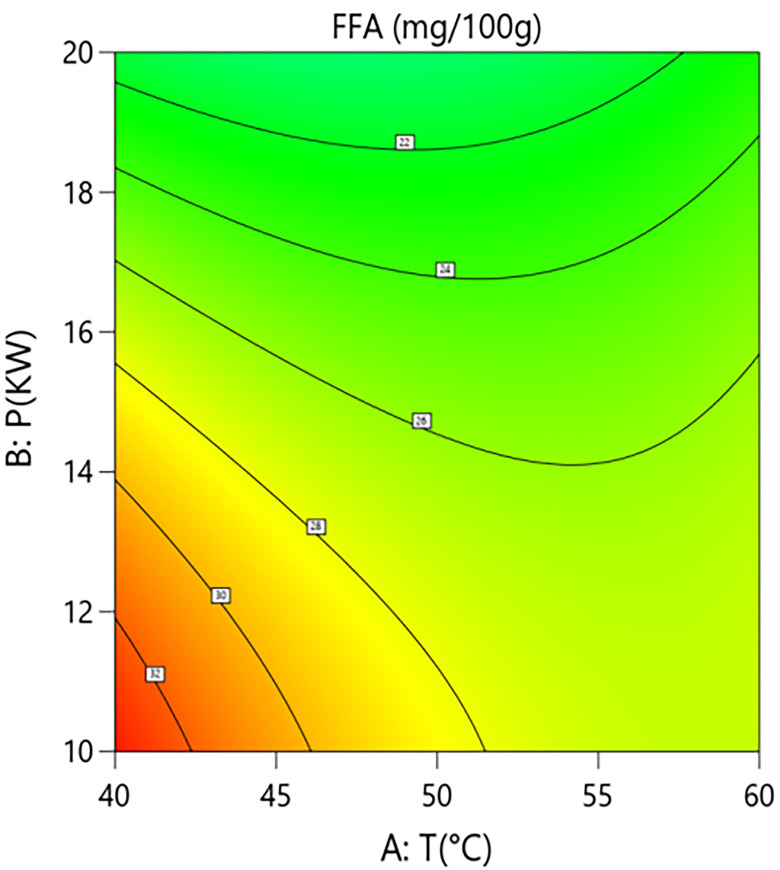
Effect of the Interaction Between Hot Air Temperature (T) and Microwave Power (P) on Free Fatty Acid Content in Rice: Contour Plot.

**Fig 15:**
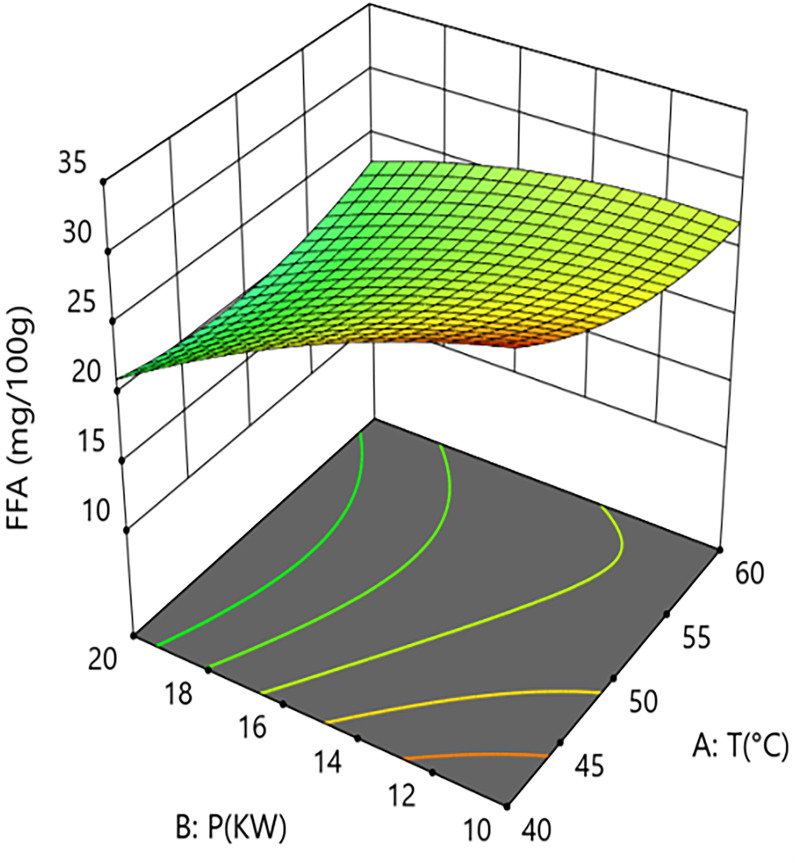
Effect of the Interaction Between Hot Air Temperature (T) and Grain Layer Thickness (h) on Calcium Content in Rice:Response Surface Plot.

**Fig 16:**
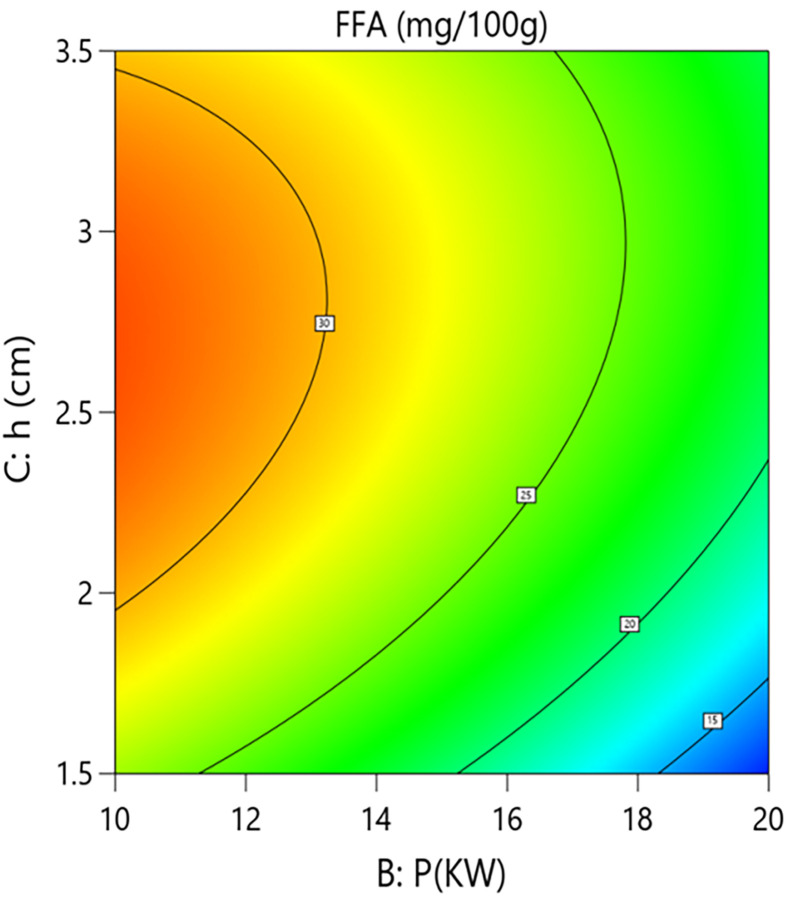
Effect of the Interaction Between Microwave Power (P) and Grain Layer Thickness (h) on Free Fatty Acid Content in Rice:Contour Plot.

**Fig 17:**
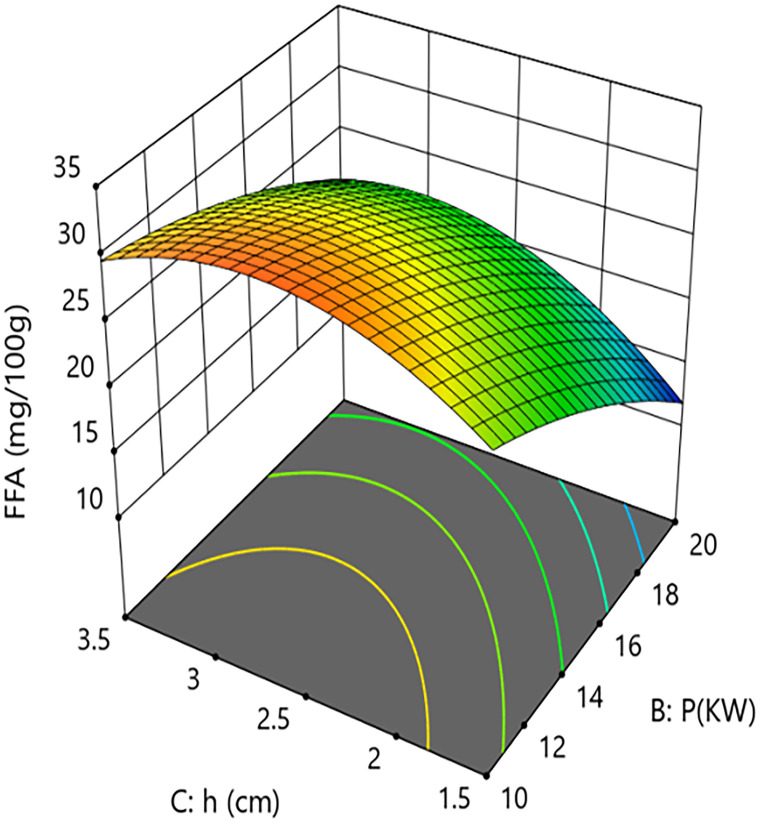
Effect of the Interaction Between Microwave Power (P) and Grain Layer Thickness (h) on Free Fatty Acid Content in Rice:Response Surface Plot.

Interaction analysis further revealed synergistic regulatory relationships between the parameters. As shown in Figs 14 and 15, the interaction term between hot-air temperature and microwave power (A × B = +2.05) had a significant effect on FFA content (P = 0.013). This positive interaction indicates that increasing the hot-air temperature weakens the inhibitory effect of microwave power on FFA formation. Response surface quantitative analysis showed that under central point grain layer thickness conditions, when the hot-air temperature was 50°C, increasing microwave power from 10 kW to 20 kW reduced FFA content from 26.1 mg/100g to 20.4 mg/100g, an absolute reduction of 5.7 mg/100g and a relative decrease of 21.8%. However, at a higher hot-air temperature level, the same increase in microwave power only reduced FFA content from 26.1 mg/100g to 22.3 mg/100g, a decrease of 14.6%. This suggests that excessively high hot-air temperatures in the microwave-hot air coupled drying system may partially counteract the inhibitory effect of high microwave power.As shown in Figs 16 and 17, the interaction term between microwave power and grain layer thickness (B × C = +1.45) was also significant (P = 0.0445). This positive interaction indicates that increasing the grain layer thickness weakens the negative effect of microwave power, and its influence exhibits significant non-linear characteristics. Under thin layer conditions (1.5 cm), increasing microwave power from 10 kW to 20 kW decreased FFA content from 22.6 mg/100g to 11.1 mg/100g, an absolute reduction of 11.5 mg/100g and a relative decrease of 50.9%. In contrast, under thick layer conditions (3.5 cm), the same power increase only reduced FFA content from 26.1 mg/100g to 20.4 mg/100g, a decrease of 21.8%. The difference between these scenarios (50.9% vs 21.8%) demonstrates that grain layer thickness significantly modulates the spatial distribution of microwave energy. Under thin layer conditions, microwave energy distributes uniformly, enabling rapid and consistent heating of the material, thereby efficiently inhibiting lipase activity. Under thick layer conditions, the skin effect of microwaves causes energy concentration at the surface and attenuation in deeper layers. The resulting accumulated heat and moisture provide sustained conditions for lipase-mediated hydrolysis, consequently weakening the overall inhibitory effect of high microwave power on FFA content. This mechanism suggests the existence of “enzyme activity residual zones” inside thick-layer materials.

In summary, to effectively suppress FFA formation, process optimization should prioritize using medium to high microwave power (15–20 kW), where equipment permits, combined with moderate hot-air temperature (50–55°C) to avoid its counteracting effect on microwave inhibition. Simultaneously, the grain layer thickness should be controlled within a thin to medium range (1.5–2.5 cm) to ensure uniform distribution of microwave energy and efficient lipase inactivation. For processing thick-layer materials, strategies such as stirring or segmented power application should be employed to eliminate internal enzyme activity residual zones.

### Multi-objective process parameter optimization and verification

To synergistically optimize the conflicting quality indicators in the paddy drying process, this study employed the desirability function method for multi-objective analysis. This method converts each response value (Yᵢ) into an individual desirability (dᵢ) ranging between 0 and 1, where dᵢ = 0 indicates a completely unacceptable response and dᵢ = 1 represents an ideal response. The overall desirability (D) is defined as the geometric mean of all individual desirability values (D = (d₁ × d₂ × ... × d_i_)^{1/n}). A value closer to 1 signifies a better comprehensive optimization effect. Based on the “nutrition priority” principle, corresponding function types and importance weights were assigned to each response target: potassium content (K) was set as a maximize target with a weight of 4; calcium content (Ca) as a maximize target with a weight of 4; vitamin B1 content (VitB1) as a maximize target with a weight of 5 (assigned the highest priority); and free fatty acid content (FFA) as a minimize target with a weight of 3.

Global optimization was performed within the set process parameter boundaries (air temperature: 40–60°C; microwave power: 10–20 kW; grain bed thickness: 1.5–3.5 cm), yielding the optimal process parameter combination: air temperature 52.47°C, microwave power 20.00 kW, and grain bed thickness 2.78 cm. Under these conditions, the predicted values for the indicators were: potassium content 3724 mg/kg, calcium content 113.7 mg/kg, vitamin B1 content 0.290 mg/100g, and free fatty acid content 21.5 mg/100g, with a corresponding overall desirability (D) of 0.869. This D value indicates that the system achieved a high degree of comprehensive optimization among the competing quality indicators. It also visually reflects the set weighting strategy: under the premise of prioritizing vitamin B1 retention, the retention of minerals (K, Ca) was effectively accommodated, and lipid hydrolysis products (reflected by FFA) were controlled within a reasonable range.

To validate the reliability of the optimization results, experimental verification was conducted under the optimal process parameters. The results, shown in Table 9, demonstrate good agreement between the experimentally predicted values and the experimentally validated values for all response indicators, with relative errors all less than 3%. This confirms the validity and accuracy of the regression model and the optimization approach developed in this study.

**Table 9:** Predicted and validated values for the optimal process parameters.

Experimental Group	K(mg/kg)	Ca(mg/kg)	VitB1(mg/100g)	FFA(mg/100g)
1	3722	113.8	0.290	21.4
2	3727	113.8	0.290	21.5
3	3703	115.0	0.289	20.7
4	3714	115.5	0.284	20.6
Mean Predicted Value	3716.5	114.5	0.288	21.1
Experimental Validation Value	3808.2	115.9	0.292	21.48
Model Error	2.47%	1.18%	1.46%	1.78%

To validate the predictive capability of the established regression model, four sets of parameter combinations were randomly generated within their respective process ranges for validation experiments. Prediction values were calculated using the regression equations, and the relative deviations between the measured and predicted values were computed. The predicted values, measured values, and relative deviations for each indicator (K, Ca, VitB1, FFA) are presented in Tables 10–13, respectively. The results show that the relative deviations for the vast majority of validation points fall within the 10% range acceptable in engineering practice. This demonstrates that the developed regression model not only possesses a high degree of statistical significance but also exhibits good engineering applicability and predictive reliability.

**Table 10:** Predicted and actual values of calcium content.

Indicator	Experimental group	T(°C)	P(KW)	h(cm)	Predicted values	Actual values	error
Ca	1	42	13	1.5	113.4	115.3	1.61%
	2	40	10	2.5	116.0	118.0	1.66%
	3	55	17	2.6	108.2	109.3	1.01%
	4	51	12	2.1	108.3	109.2	0.82%

**Table 11:** Predicted and actual values of free fatty acid content.

Indicator	Experimental group	T(°C)	P(KW)	h(cm)	Predicted values	Actual values	error
FFA	1	41	14	2.5	29.3	30.3	3.27%
	2	40	10	2.5	32.9	34.1	3.46%
	3	57	19	3.2	23.8	24.6	3.13%
	4	54	18	1.7	16.7	17.6	4.89%

**Table 12:** Predicted and actual values of potassium content.

Indicator	Experimental group	T(°C)	P(KW)	h(cm)	Predicted values	Actual values	Error
K	1	44	11	1.6	3690	3580	−3.07%
	2	48	17	1.9	3580	3670	2.45%
	3	57	18	2.8	3630	3700	1.89%
	4	40	10	2.5	3650	3770	3.18%

**Table 13:** Predicted and actual values of vitamin B1 content.

Indicator	Experimental group	T(°C)	P(KW)	h(cm)	Predicted values	Actual values	Error
VitB1	1	49	10	1.5	0.268	0.276	2.9%
	2	58	20	1.9	0.307	0.291	−5.4%
	3	40	10	2.5	0.261	0.269	2.97%
	4	53	18	3.1	0.288	0.279	−3.22%

### Quality process reference diagrams

Building upon our team’s previously established process reference maps for rice drying [21,22,33], this study developed a multi-objective co-optimization process reference map by integrating drying characteristics, processing parameters, and nutritional quality indicators. This map can predict the values of specific quality indicators in dried rice under different parameter combinations. It also aids engineers in understanding the comprehensive impact of parameter adjustments on multiple key quality indicators, thereby facilitating informed trade-off decisions.

**Fig 18:**
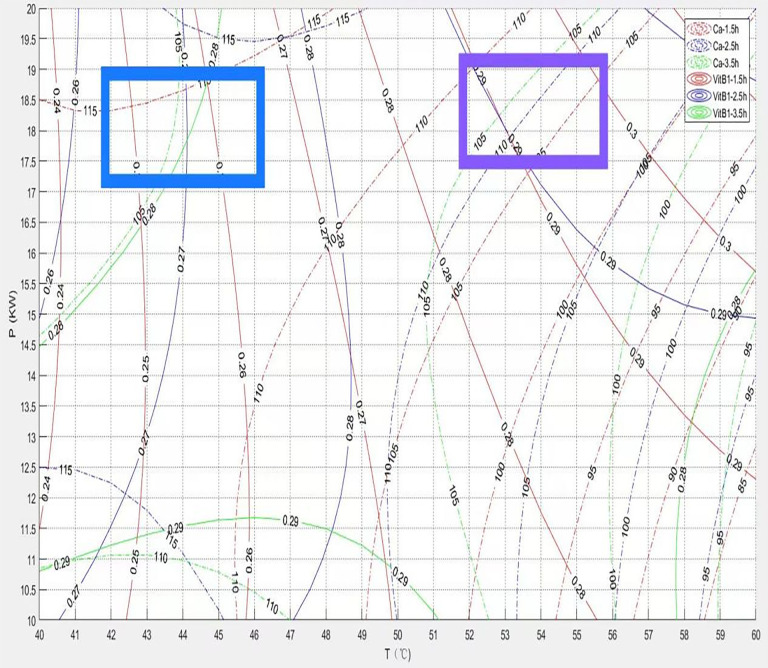
Process reference diagram coupling calcium and vitamin B1 contents.

Fig 18 displays the process reference map integrating Calcium (Ca) and Vitamin B1 (VitB1) retention rates. Analysis reveals an intrinsic conflict between achieving high Ca retention and high VitB1 retention. High temperatures significantly accelerate the thermal degradation of Vitamin B1 (via oxidation and Maillard reactions), leading to a decrease in VitB1 retention. In contrast, calcium, as a mineral element, maintains relatively stable chemical forms at high temperatures and is not prone to direct thermal decomposition. High temperatures enhance drying efficiency, reducing physical losses caused by moisture migration. Simultaneously, they may alter the material structure (e.g., promoting starch gelatinization or protein cross-linking), potentially enhancing the stability of bound calcium or its degree of fixation in the final product, thereby favoring Ca retention.While low temperatures help protect the heat-sensitive Vitamin B1, they prolong drying time and reduce production efficiency. A low drying rate may increase the risk of water-soluble calcium migrating to the surface with moisture and being lost, indirectly affecting calcium stability or bioavailability.The blue-shaded area in the diagram represents the low-temperature operation zone where vitamin B1 retention is high. Its core strategy lies in minimizing thermal damage, which is the key factor for protecting VB1. For example, choosing fortified rice exemplifies this strategy. The purple-shaded area corresponds to the medium-high temperature operation zone where calcium retention is high. Higher temperatures facilitate rapid dehydration and promote the stabilization of minerals; simultaneously, calcium itself possesses good thermal stability. This zone focuses on optimizing the content of minerals.

**Fig 19:**
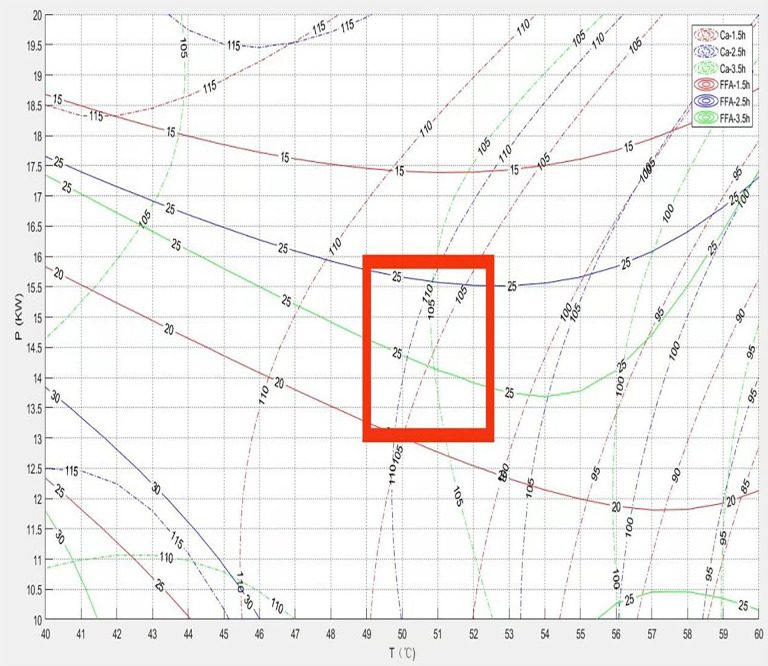
Process reference diagram coupling calcium and free fatty acid contents.

Fig 19 shows the process reference map integrating Calcium (Ca) retention rate and Free Fatty Acid (FFA) content. At higher initial grain layer thicknesses, Ca retention rate initially increased and then decreased as temperature rose, while FFA content showed a slight initial decrease followed by a significant increase. Increasing temperature enhances drying efficiency and accelerates moisture removal. Simultaneously, moderate heat treatment (e.g., starch gelatinization or protein denaturation) can form a more stable matrix microstructure, fixing minerals and thereby increasing calcium retention. However, excessively high temperatures accelerate the Maillard reaction; the resulting complex compounds can alter the mineral environment, reducing its stability and leading to a decline in Ca retention. For free fatty acids, the initial temperature rise may inhibit FFA generation by rapidly reducing water activity and partially inactivating enzymatic activity. However, a continued temperature increase damages cell membranes or liposome structures, exposing internal lipids fully to oxygen, exacerbating lipid oxidation, and causing a significant rise in FFA content. Fig 11 clearly indicates that thick grain layers constitute a significant risk factor for process stability and product quality. In practical operations, it is necessary to establish and strictly adhere to a safe upper limit for grain layer thickness, while optimizing airflow distribution and microwave field uniformity to minimize heating non-uniformity and prevent severe deterioration in localized areas. The zone delineated by the red line in the figure represents the optimal parameter combination interval for achieving the dual objectives of low FFA (indicating good storage stability) and high calcium retention. This zone represents a balance point between nutrient retention (high Ca retention) and oil stability (low FFA).

**Fig 20:**
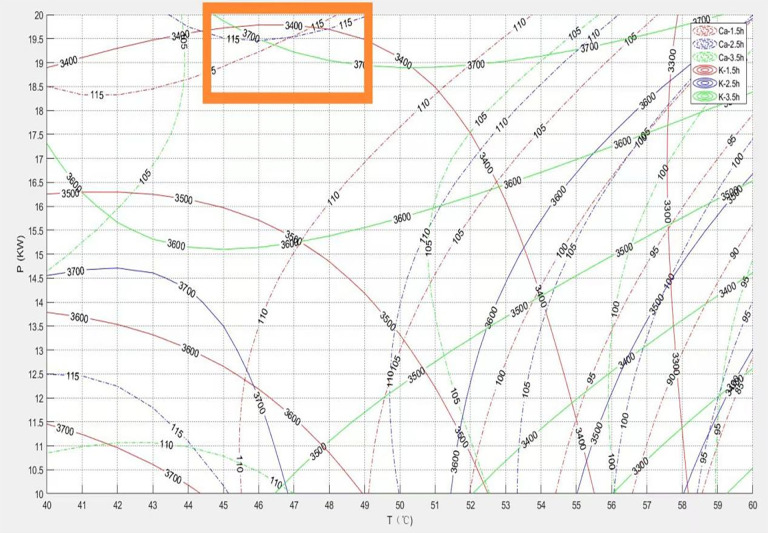
Process reference diagram coupling calcium and potassium contents.

Fig 20 displays the process reference map integrating Calcium (Ca) and Potassium (K) retention rates. Calcium retention showed a slight downward trend as grain layer thickness increased, with a relatively small magnitude of change, indicating that Ca retention is more sensitive to temperature than to grain layer thickness. Potassium retention, however, was more sensitive to changes in grain layer thickness: in the thin layer region (low h), potassium retention decreased; as layer thickness increased, potassium retention rose; entering the medium-to-high layer thickness region, the growth rate of potassium retention slowed and tended towards relative stability.Under thin layer conditions, the intense rapid dehydration process caused significant amounts of moisture, containing dissolved potassium ions, to rapidly migrate to the material surface and be lost, resulting in severe potassium depletion. Calcium loss was relatively smaller and more gradual in this region, making potassium retention the critical limiting factor and optimization target. Under thick layer conditions, moisture evaporation slows, weakening potassium loss via moisture migration. However, excessively thick layers may cause localized overheating or prolong exposure to a hot-humid environment, increasing the risk of calcium ion loss due to structural damage, adverse chemical reactions, or slow migration. Consequently, the relative importance of calcium preservation increases in this region. The region indicated by the orange line in the figure corresponds to the optimal parameter combination where both potassium and calcium retention rates achieve relatively high levels, effectively balancing both requirements.

## Conclusion

This study systematically elucidates the regulation mechanisms of microwave-assisted drying parameters (T, P, h) on key nutritional components (K, Ca, VitB1) and storage stability indicators (FFA) in paddy rice using a multi-factor response surface methodology. The quadratic regression models constructed for all response variables demonstrated high statistical significance (P < 0.01) and revealed a synergistic degradation effect of the T*P interaction term on vitamin B1 within the electro-thermal coupling field, thereby providing deeper thermodynamic insights into the redox reaction mechanisms during drying. Through model optimization and experimental validation (relative error < 3%), the optimal parameter combination (T = 52.47°C, P = 20 kW, h = 2.78 cm) was determined. Based on this, visualized process reference maps were developed, offering practical guidance for low-cost and precise process decision-making, which holds significant reference value for developing intelligent drying systems capable of adaptive parameter adjustment.However, this study and its models are subject to specific boundaries and limitations: the model validity is strictly confined to the experimental parameter ranges, requiring cautious extrapolation beyond these limits; the construction of simplified response surfaces focused on three key parameters while excluding practical factors such as ambient humidity and rice variety variations, potentially introducing bias in more complex real-world applications; furthermore, the optimization targets were confined to specific quality indicators, whereas simultaneous consideration of drying efficiency and energy consumption would necessitate more sophisticated multi-objective optimization frameworks. These limitations pose substantial challenges in industrial translation, particularly regarding the uniformity of hot air distribution, stability of microwave field intensity, and dynamic control of material handling in industrial-scale equipment. Consequently, the development of adaptive control systems based on such process maps is crucial to bridge the gap from laboratory-scale optimization to large-scale application.Building upon these findings, future research should extend the existing “modeling-optimization-visualization” framework to various paddy rice varieties and other cereals to validate its universality and establish a comprehensive process database. Subsequent efforts should focus on multi-objective Pareto optimization to balance quality, efficiency, and energy consumption, thereby identifying Pareto-optimal solutions. Further exploration should integrate static process maps with online real-time detection technologies to develop dynamic feedback control strategies, ultimately advancing toward fully intelligent drying processes. The methodological framework established herein is not limited to grain drying and can be extended to other industrial processes requiring precise parameter optimization for quality control, such as food processing and pharmaceutical manufacturing.

## Supporting information

S1 File The procedure required for the correlation between calcium content and vitamin B1 content. (DOCX)

S2 File The procedure required for the correlation between calcium content and free fatty acid content. (DOCX)

S3 File The procedure required for the correlation between calcium content and potassium content. (DOCX)
