# Supplementary material for: Process optimization of microwave drying for rice based on response surface methodology
Source: PLoS One. 2026 Jan 13;21(1):e0340356. doi: 10.1371/journal.pone.0340356 (PMC12799006; doi:10.1371/journal.pone.0340356)
Supplement: S3 File — (DOCX) [file pone.0340356.s003.docx]

**The procedure required for the correlation between calcium content and potassium content.**

figure ('NAME')

x=40:1:60;

y=10:0.5:20;

[X,Y]=meshgrid(x,y);

xlabel('T（℃)');

ylabel('P (KW)');

title('');

set(gca,'xtick',40:1:60);

set(gca,'ytick',10:0.5:20);

grid on;

set(gca,'GridLineStyle',':','GridColor','k','GridAlpha',1);

hold on;

Z1=93.50625+2.39958*X-4.9275*Y+10.12917*1.5+0.0895*(X.*Y)+0.3925*X*1.5-0.55*Y*1.5-0.055583*(X.^2)+0.076667*(Y.^2)-4.33333*1.5^2;

Z2=93.50625+2.39958*X-4.9275*Y+10.12917*2.5+0.0895*(X.*Y)+0.3925*X*2.5-0.55*Y*2.5-0.055583*(X.^2)+0.076667*(Y.^2)-4.33333*2.5^2;

Z3=93.50625+2.39958*X-4.9275*Y+10.12917*3.5+0.0895*(X.*Y)+0.3925*X*3.5-0.55*Y*3.5-0.055583*(X.^2)+0.076667*(Y.^2)-4.33333*3.5^2;

contour(X,Y,Z1,[85 90 95 100 105 110 115 120],'-.R','showtext','on');

%contour(x,y,z,v,);

contour(X,Y,Z2,[85 90 95 100 105 110 115 120],'-.B','showtext','on');

contour(X,Y,Z3,[85 90 95 100 105 110 115 120],'-.G','showtext','on');

Z4=4458.125+22.5*X-179.5*Y+132.5*1.5+2.2*(X.*Y)+4*X*1.5+24*Y*1.5-0.775*(X.^2)+0.5*(Y.^2)-127.5*1.5^2;

Z5=4458.125+22.5*X-179.5*Y+132.5*2.5+2.2*(X.*Y)+4*X*2.5+24*Y*2.5-0.775*(X.^2)+0.5*(Y.^2)-127.5*2.5^2;

Z6=4458.125+22.5*X-179.5*Y+132.5*3.5+2.2*(X.*Y)+4*X*3.5+24*Y*3.5-0.775*(X.^2)+0.5*(Y.^2)-127.5*3.5^2;

contour(X,Y,Z4,[3200 3300 3400 3500 3600 3700 3800],'R','showtext','on');

contour(X,Y,Z5,[3200 3300 3400 3500 3600 3700 3800],'B','showtext','on');

contour(X,Y,Z6,[3200 3300 3400 3500 3600 3700 3800],'G','showtext','on');

legend(('Ca-1.5h','Ca-2.5h','Ca-3.5h','K-1.5h','K-2.5h','K-3.5h')%
